# Involvement of chemokine CXCL12 and its receptor CXCR4 in uterine receptivity and potential relationship to fertility in cattle: a mini review

**DOI:** 10.3389/fvets.2025.1651593

**Published:** 2025-08-29

**Authors:** Michael J. D’Occhio, Giuseppe Campanile, Pietro S. Baruselli

**Affiliations:** ^1^School of Life and Environmental Sciences, Faculty of Science, The University of Sydney, Sydney, NSW, Australia; ^2^Department of Veterinary Medicine and Animal Production, University of Naples Federico II, Naples, Italy; ^3^Department of Animal Reproduction, Faculty of Veterinary Medicine and Animal Science, University of São Paulo, São Paulo, Brazil

**Keywords:** CXCL12, CXCR4, cow, embryo, uterus, receptivity, fertility

## Abstract

The establishment of a pregnancy in cattle relies on crosstalk between an embryo with high developmental competence and a responsive uterus. This often fails and the pregnancy rate in cattle is around 60–70% with natural mating and 50–60% for embryo transfer, with pregnancies typically higher in beef than high performing dairy. These pregnancy rates are primarily due to the loss of embryos in the 21-day window from fertilization to the initiation of attachment of the conceptus to the uterus. Considerable research has been devoted to defining high quality embryos; however, embryonic mortality remains a major cause of pregnancy failure. The latter highlights the critical importance of uterine receptivity in establishing a pregnancy. The uterus must be responsive to signals from the developing embryo to undergo a major structural and functional transformation to prepare for attachment of the conceptus and establishment of pregnancy. The chemokine CXCL12 and its receptor CXCR4 are expressed across somatic and neural tissues and are associated with tissue remodeling including angiogenesis. These are features of the change the uterus undergoes as it develops receptivity to the conceptus. The developing embryo produces CXCL12 and CXCR4 is present in uterine tissue, and a role for the CXCL12-CXCR4 axis have been demonstrated in early pregnancy. Chemokines including CXCL12 are likely to be important in embryonic survival and pregnancy in cattle.

## 1 Background

Pregnancy rates in cattle following natural mating or with assisted reproductive technology have remained relatively constant at around 60–70% with natural mating and 50–60% for embryo transfer, with pregnancies typically higher in beef than high performing dairy (1–3). The primary reason why pregnancy rates have not improved as might have been expected is the failure to overcome the large embryonic loss that occurs in the period before and during the attachment of an embryo to the uterus to establish a pregnancy (2, 4–6). The period of early embryonic development involves continuous crosstalk between the embryo and uterus (7, 8). The embryo initiates this crosstalk by secreting interferon tau (IFNτ) which prevents the uterus from generating an immune response against the allogeneic embryo (9–12). Interferon tau-stimulated gene expression in blood mononuclear cells was evaluated as a biomarker of early pregnancy in cattle (13–16). The developing embryo also secretes factors that induce changes in the structure and function of the uterus, which prepares the uterus for embryonic attachment (16–20). The preparation of the uterus for attachment confers uterine receptivity (21, 22). Chemokines and their receptors have an important role in this process and an example is the embryonic chemokine ligand stromal-derived factor 1α (CXCL12) which binds to its uterine receptor CXCR4 (23). As noted below, *CXCL12* and *CXCR4* are expressed across somatic and neural tissues and are associated with tissue remodeling including angiogenesis. These are features of changes the uterus undergoes as it develops receptivity to the conceptus. The present review draws on information for cytokines and their receptors and in particular CXCL12-CXCR4 in female reproduction in several species to highlight the need for further research in cattle. A potential outcome of further research could be the identification of *CXCL12-CXCR4* gene polymorphisms that are linked to uterine receptivity and fertility in cattle (24). This would require the collection of phenotypic information on large cohorts of cattle to achieve statistical power to identify meaningful polymorphisms. Given fertilization and formation of a zygote in cattle is typically greater than 75%, we have argued that the next step change in reproductive success in cattle will require a reduction in embryonic loss with both natural mating and assisted reproductive technology (10, 20, 25, 26).

## 2 Female effect on fertility in cattle

The capacity of female cattle to conceive and wean a calf on an annual basis is the primary driver of profitability in cattle enterprises (27). As noted above, fertilization rates in cattle are typically greater than 75% with both natural mating and artificial insemination (25). Fertilization *per se* is therefore not the major reason for reproductive failure in cattle. The main cause of reproductive failure in cattle, and indeed females of other species, is the large loss of embryos that occurs in the 21-day window from fertilization to the initiation of attachment of the embryo to the uterus (20, 25, 28, 29). Embryonic survival was identified early as arguably the most important factor in determining pregnancy outcome in cattle (30–32). In one study, a significant recipient effect was observed in pregnancy rate when Hereford x Friesian heifers received six cycles of embryo transfer (31). Heifers retrospectively classified as ‘high fertility’ had an overall pregnancy rate of 76% and heifers classified as ‘low fertility’ had a pregnancy rate of 11% (31). At day 14 after embryo transfer, more embryos had undergone elongation in ‘high fertility’ heifers (67%) compared with ‘low fertility’ heifers (14%) (31). The heifer effect was noticeable during the period of embryonic attachment and pregnancy establishment, with no apparent effect after day 60 when the determination of the effect was diminished (31, 32). In another study also involving serial embryo transfer, beef heifers classified ‘high fertile’ showed a pregnancy rate of 71% compared with a pregnancy rate of 20% for heifers classified ‘infertile’ (33). Similar with the earlier study in dairy heifers, elongating conceptuses were longer in ‘high fertile’ beef heifers compared with ‘infertile’ heifers (33). ‘High fertile’ heifers showed greater uterine expression of genes associated with conceptus-uterus crosstalk which was interpreted to indicate that ‘high fertile’ heifers had a greater capacity to support conceptus growth, attachment and pregnancy (33). Studies in Holstein cows led to the conclusion that the difference in fertility between ‘high fertile’ (Fert+) and ‘low fertile’ (Fert-) cows was related to embryonic and uterine events after day 7, which likely included the capacity of cows to support ongoing embryonic development, attachment and pregnancy (34). In the above studies, oocytes and embryos from high and low fertile females did not differ in gene expression and other functional parameters providing further evidence of the importance of the uterine response to the embryo in pregnancy (4, 33, 34). Pregnancy does, however, rely on the combination of a good quality embryo with high developmental competence and a responsive uterus (4).

## 3 Uterine (endometrial) receptivity

The capacity of the uterus to support attachment of the conceptus, followed by the events that establish a pregnancy, relies on uterine (endometrial) receptivity irrespective of the type of placentation. The change from a non-receptive to receptive uterus occurs in response to the conceptus and involves major changes in uterine structure and function (16–18, 35, 36). The endometrium in cattle undergoes a major change in preparation for embryonic attachment and pregnancy (8, 20). The ovarian steroids oestradiol and progesterone induce initial changes in the uterine endometrium in cattle and further change is a result of ‘mutual reprogramming’ between the conceptus and uterus (21, 22). Changes in endometrial gene expression around day 15 in cattle are induced by embryonic IFNτ (16). The application of machine learning identified endometrial transcriptomic biomarkers that predicted uterine receptivity with around 95% accuracy in cattle (37, 38). The latter suggested that establishing uterine receptivity through a uterine biopsy could potentially be used as a fertility trait in cattle (38, 39). Embryos also induce changes in uterine fluid microRNAs and exosomes in cattle (40, 41). Uterine receptivity has been extensively studied in women to more precisely define the ‘implantation window’ in conjunction with efforts to increase the efficiency of IVF and embryo transfer (42–46). In Mediterranean buffaloes, the period of implantation is associated with changes in blood flow and capillary permeability of uterine caruncles (47, 48).

## 4 Chemokines and their receptors

Chemokines are a family of chemoattractant cytokines that have important roles in cell migration and angiogenesis (49–51). Cell differentiation and migration, and angiogenesis, are central to tumor metastasis and a large body of literature describes the role of CXCL12 in conditioning stromal cells for invasion by cancer cells (52–58). Stromal-derived factor 1α (CXCL12) is an important chemokine that is expressed in both somatic and neural tissues (52, 59–61). The receptor for CXCL12, CXCR4, is also widely distributed in somatic and neural tissues (53, 55, 62). Most studies on CXCR4 have been in cancer biology and other diseases (50, 52–55, 61, 63–66). CXCL12 can also bind to the orphan receptor CXCR7 (ACKR3) which functions as a scavenger and could have a role in the local actions of CXCL12 (56).

Both CXCL12 and CXCR4 have been characterized at the genomic and protein level. In cattle, the gene *CXCL12* is identified as ENSBTAG00000005077 (primary assembly *Bos taurus* genome, ARS-UCD2.0) and is located at base-pair position 28:45021867–45052552^1^. The gene has two variants each of which contains four exons. ENSEMBL identifiers for the transcripts are ENSBTAT00000015300.1 (CXCL12-201) and ENSBTAT00000031279.5 (CXCL12-202). Cattle *CXCR4* is tagged ENSBTAG00000001060, is located at 2:612249996–61254590, and has three transcripts and also splice variants^2^. The human *CXCL12* gene is located at 10q11.1 and the promoter region has binding sites for the transcription factors SP1 and CTF (52, 60, 66). *CXCL12* is unique among CXC chemokines in that it has differential mRNA splicing with six splice variants which give rise to six different isoforms in humans, with three isoforms in mice (60). Both the *CXCL12* gene and protein show high (90%) homology between humans and mice (60). Typical CXCL12 protein is relatively small with 68 amino acids (52). The *CXCR4* gene is located at human 2q21 and the CXCR4 protein has 352 amino acids (64, 65). CXCR4 is a G protein-coupled receptor and signaling/transducing pathways include mammalian target of rapamycin (mTOR), phosphoinositol 3 kinase/protein kinase B and Janus kinase/signal transducers and activators of transcription (JAK/STAT), among other pathways (52, 64, 65).

## 5 CXCL12 and CXCR4 in uterine remodeling and receptivity

**Figure 1:**
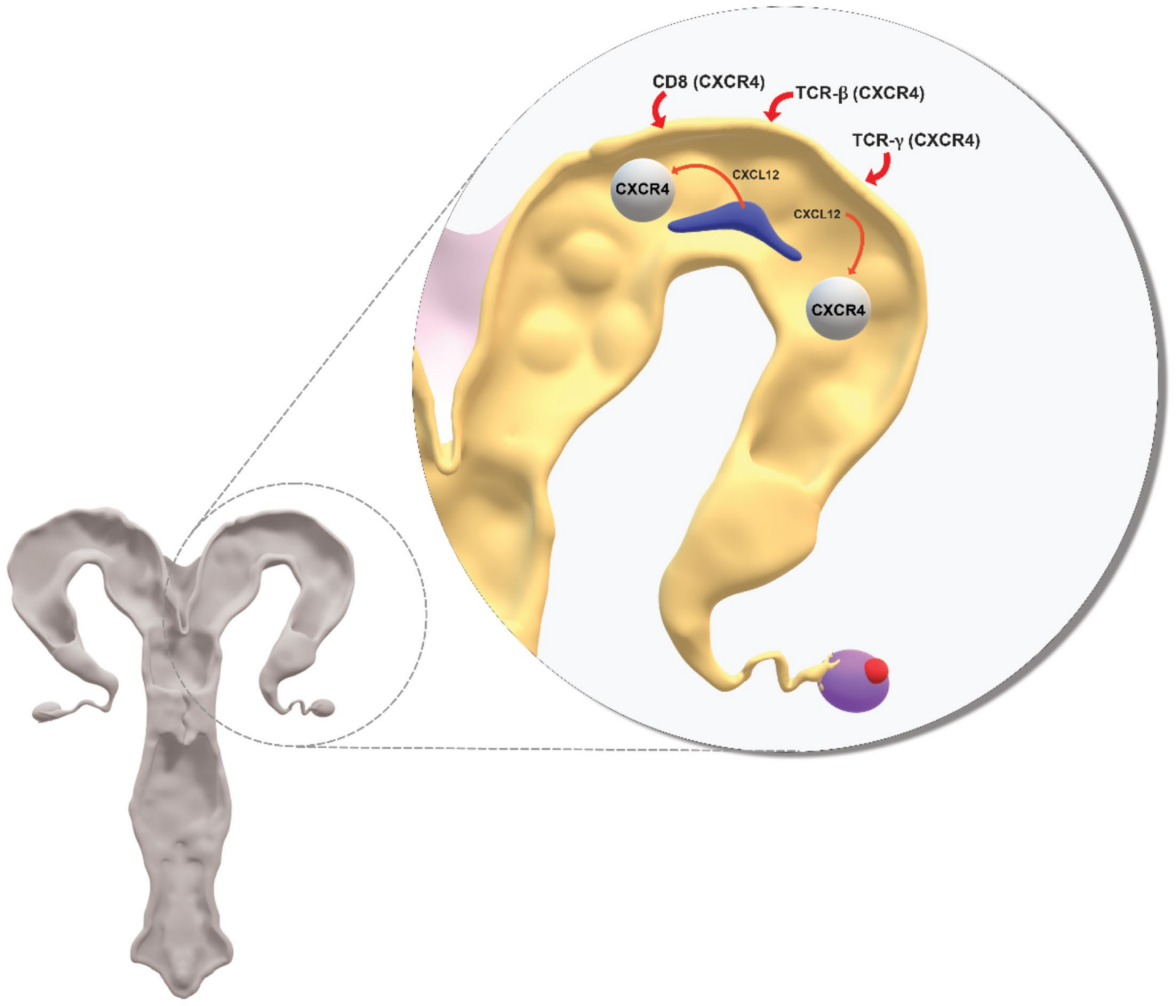
Conceptual diagram on role of CXCL12-CXCR4 in crosstalk between the conceptus and uterus during the period when the uterine endometrium undergoes major structural and functional change in preparation for embryo attachment to the epithelium, implantation and pregnancy. CXCL12 secreted by the conceptus acts at its CXCR4 receptor to induce changes at the uterus. CXCL12 additionally acts at CXCR4 receptors on immune cells (CD8, TCR-*β*, TCR-*γ*) recruited from blood and which are involved in inflammatory processes associated with the establishment of uterine receptivity. Support for the model of CXCL12-CXCR4 is in the cited literature.

The uterine epithelium and stroma undergo major cellular reorganization in response to the presence of an embryo and in preparation for attachment, implantation, and the establishment of a pregnancy (7, 21, 36, 45, 46). Chemokines are now recognized as having an important role in the changes that occur in the uterine endometrium during the period before attachment of the conceptus (23, 67–69). The C-C and CXC-motif chemokines were shown to influence endometrial epithelial cell function, implantation and embryo survival in cattle (70–76). In humans, CXCL12 is produced by embryonic trophoblast cells and induces uterine stromal cells to express its receptor CXCR4 (77, 78). Both *CXCL12* and *CXCR4* are expressed in uterine endometrial epithelial cells and stromal cells and are considered to have an important autocrine role in remodeling of the epithelium in preparation for attachment of the conceptus (Figure 1) (23, 67–69). CXCL12-CXCR4 facilitated infiltration of the uterus by natural killer (NK) cells which is part of the immune cell remodeling of the epithelium and stroma mice (79). *CXCR4* knock-out mice had reduced NK cells and increased fetal resorption and significantly reduced implantation (78). CXCL12 obtained from pre- and peri-implanting mice increased angiogenesis and embryo attachment in *in vitro* cultures of mouse tissues (80). Treatment with CXCL12 induced CXCR4^+^ Treg cells to infiltrate the uterus and create a supportive environment for attachment and pregnancy in a diabetic mouse model (81). The CXC chemokines have been implicated in the pathology of endometritis in women but this field is outside the scope of the present article (82, 83).

Ewes treated with the CXCR4 antagonist AMD3100 from day 12 to day 20 after breeding had diminished uterine levels of angiogenic factors which demonstrated the role of CXCL12-CXCR4 in vascularisation of the utero-placental unit (84). In a second study of similar design in sheep, treatment with the antagonist AMD3100 from day 12 to day 35 after breeding was associated with increased autophagy induction at the fetal-placental unit (85). Also in sheep, intra-uterine treatment with antagonist AMD3100 from day 7 to day 14 after mating resulted in abnormal placental function (86). In a further study in sheep, expression of *CXCL12* and *CXCR4* were increased in conceptus and uterus around the time of attachment and placentation (87). *CXCL12* expression in trophoblast and endometrial stroma of sheep was greater in natural mated ewes compared with ewes that received IVF embryos (88). The expression of *CXCL12* in endometrial stroma was interpreted to indicate that CXCL12 can have a paracrine and/or autocrine action (88). CXCL12 and CXCR4 were reported to be associated with luminal epithelial cell remodeling in pigs (69, 89). In cattle, *CXCR4* mRNA in endometrium did not change from day 14 to day 50 in pregnant cows (90). CXCR4 mRNA was, however, increased in blood on day 20 to day 32 which coincided with the period of implantation in cattle. A secondary increased in blood CXCR4 mRNA from day 30 coincided with caruncular-cotyledonary placentome development in cattle. mRNA for immune cells CD8, TCR-*β* and TCR-*γ* was increased in blood and mRNA for CD8 and TCR-β was increased in endometrium on day 19 (90). It was proposed that blood-derived immune cells that express *CXCR4* populate the uterus and are involved in uterine inflammation associated with embryo attachment, vascularisation and placentome formation in cattle (90).

## 6 Summary

Fertilization rates in female cattle are typically greater than 75% with both natural mating and artificial insemination. The lack of fertilization *per se* is therefore not the major reason for reproductive failure in cattle. The main cause of reproductive failure in cattle, and indeed females of other species, is the large loss of embryos which occurs in the 21-day window from fertilization to attachment of the embryo to the uterus. As noted above, the establishment of a pregnancy relies on the combination of a good quality embryo with high developmental competence and a responsive uterus. This mini review has brought together information which highlights the important role of uterine receptivity in embryonic survival. A greater understanding of uterine receptivity is necessary for a meaningful step change in reproductive success in cattle. This could include studies involving endometrial biopsies in early stages of pregnancy for transcriptomic and proteomic profiling, linked with genotyping. This approach would however require significant resources. MicroRNAs are now known to regulate pathways associated with uterine receptivity and the interaction with CXCL12-CXCR4 is a further area of research (91). In a recent study, polymorphism in a region in proximity to the *CXCR4* gene was suggested as a putative causal variant for fertility in highly fertile Brahman cattle (24). This was consistent with a role for CXCL12-CXCR4 in uterine receptivity and fertility in cattle. There is a clear need to undertake mechanistic studies to demonstrate a role for the CXCL12-CXCR4 axis in uterine receptivity in cattle. There is also a need for large phenotype-genome/proteome studies to identify additional polymorphisms in the *CXCL12-CXCR4* genes and other genes associated with uterine receptivity and fertility in cattle.

## Footnotes

^1^ https://asia.ensembl.org/Bos_taurus/Location/View?r=28%3A45021867-45052552;www.cattlegeneatlas.roslin.ed.ac.uk

^2^ https://asia.ensembl.org/Bos_taurus/Gene/Splice?db=core;g=ENSBTAG00000001060;r=2:61250084-61254502
